# T2-mapping in normal volunteers compared to patients with edema reveals wide range of myocardial T2 in female volunteers

**DOI:** 10.1186/1532-429X-14-S1-O81

**Published:** 2012-02-01

**Authors:** Ralf Wassmuth, Andreas Greiser, Jeanette Schulz-Menger

**Affiliations:** 1 Cardiology, Helios Klinikum and Charite University Medicine Berlin, Berlin, Germany; 2 Imaging and IT Division, Siemens Healthcare, Erlangen, Germany

## Summary

We applied T2 mapping in 72 volunteers compared to 15 patients with myocardial edema. T2 of myocardial edema was 70 ms, whereas normal myocardium had a T2 of 55ms. However, female volunteers showed a wider range of myocardial T2 with overlap into values considered abnormal.

## Background

T2-mapping has been proposed to overcome limitations of T2-weighted imaging of myocardial edema. We applied T2-mapping in normal volunteers to reveal the inter- and intra-individual homogeneity of myocardial T2 and to compare it to patients with myocardial edema.

## Methods

We scanned 72 healthy volunteers (36±13 years, 18 females, heart rate 71±10 bpm) in a 1.5 T scanner. All had no cardiac disease and no signs of inflammation. We applied a FLASH and a SSFP-based T2-mapping sequence resulting in 3 single-shot images with echo times of 0, 24 and 54 ms and a pixel size of 2.7 x 2.1 mm (slice thickness 8 mm). Images were obtained in midventricular short axis and 4-chamber-view (4CV). We measured global and segmental T2 according to a 16-segment model. Contours were manually drawn on images by two independent observers and then copied to the map. 26 volunteers underwent additional mapping with reduced pixel size of 2.2 x 1.8 mm and automatic motion correction. 6 volunteers were scanned twice on separate days. Results were compared to 15 patients with myocardial edema (12 infarctions, 1 myocarditis, 1 takotsubo, 1 sarcoidosis).

## Results

We excluded 4 volunteers due to obvious pathologies. In patients myocardial edema had a T2 of 71±4 ms (p<0.001 compared to volunteers). In volunteers mapping based on FLASH resulted in lower T2-values than SSFP (52±4 vs. 55±5 ms; p< 0.001). T2 values were independent from heart rate, age or body weight. Apical septal segments had higher values than basal lateral segments in 4CV (64±8 vs. 55±5 ms for SSFP, p< 0.001). Women below 36 years had a 95% confidence interval of 43-86 ms based on SSFP mapping on 4CV images.

Mean intraobserver variability was 0.8±0.9 ms (correlation coefficient r=0.99), mean interobserver variability was 1.6±1.8 ms (correlation coefficient r=0.98). Variability for repeated scans was 2.3±1.8 ms. Improved spatial resolution and the applied motion correction did not significantly change the T2-measurements (p=0.3).

## Conclusions

T2 mapping is feasible with low intra- and interobserver variability. T2 of myocardial edema was 70 ms, whereas normal myocardium had a T2 of 55ms. However, female volunteers showed a wider range of myocardial T2 with overlap into values considered abnormal. This is most likely due to myocardial contamination with blood (T2 of about 180 ms). Motion correction and spatial resolution needs to be improved before T2-mapping can widely be applied for exclusion of edema.

## Funding

The scanner manufacturer provided a preliminary version of the mapping sequence based on the local research agreement. Funding was provided by the university.

**Figure 1:**
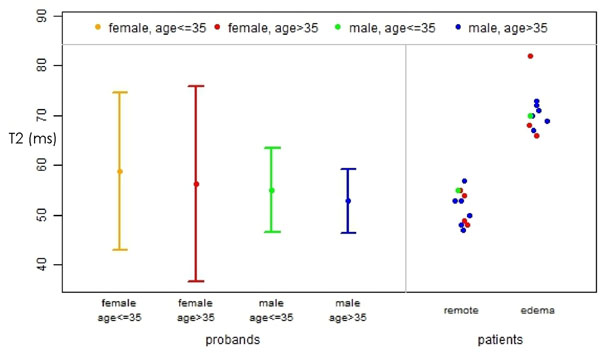
Mean myocardial T2 and 95% CI for female and male volunteers (left) compared to patients with edema (right).

